# Clinical effectiveness of symptomatic therapy compared with standard step-up care for the treatment of low-impact psoriatic oligoarthritis: the two-arm parallel group randomised POISE feasibility study

**DOI:** 10.1177/1759720X211057668

**Published:** 2022-01-10

**Authors:** Ines Rombach, Laura Tucker, William Tillett, Deepak Jadon, Marion Watson, Anne Francis, Yvonne Sinomati, Susan J Dutton, Laura C Coates

**Affiliations:** Oxford Clinical Trials Research Unit, Nuffield Department of Orthopaedics, Rheumatology and Musculoskeletal Sciences, University of Oxford, Oxford, UK; Nuffield Department of Orthopaedics, Rheumatology and Musculoskeletal Sciences, University of Oxford, Oxford, UK; Royal National Hospital for Rheumatic Diseases, Bath, UK; Department of Pharmacy & Pharmacology, University of Bath, Bath, UK; Department of Medicine, University of Cambridge, Cambridge, UK; Oxford Clinical Trials Research Unit, Nuffield Department of Orthopaedics, Rheumatology and Musculoskeletal Sciences, University of Oxford, Oxford, UK; Oxford Clinical Trials Research Unit, Nuffield Department of Orthopaedics, Rheumatology and Musculoskeletal Sciences, University of Oxford, Oxford, UK; Oxford Clinical Trials Research Unit, Nuffield Department of Orthopaedics, Rheumatology and Musculoskeletal Sciences, University of Oxford, Oxford, UK; Oxford Clinical Trials Research Unit, Nuffield Department of Orthopaedics, Rheumatology and Musculoskeletal Sciences, University of Oxford, Oxford, UK; Botnar Research Centre, Nuffield Department of Orthopaedics, Rheumatology and Musculoskeletal Sciences, University of Oxford, Windmill Road, Oxford OX3 7LD, UK

**Keywords:** clinical trial, oligoarthritis, psoriatic arthritis

## Abstract

**Introduction:**

In psoriatic arthritis (PsA), treatment recommendations support first-line use of disease-modifying antirheumatic drugs (DMARDs). There are few treatment strategy trials, and no previous studies have investigated tailored treatment choice by disease severity. Studies in oligoarthritis (<5 inflamed joints) are limited but have suggested that some can be managed without DMARDs, preventing unnecessary side effects. This study aimed to assess the feasibility and acceptability of a study comparing standard DMARD treatment against symptomatic therapy in patients with mild psoriatic oligoarthritis.

**Methods:**

This trial was embedded within the MONITOR-PsA cohort, which uses a Trials Within Cohorts (TWiCs) design. Patients with newly diagnosed psoriatic oligoarthritis, with low disease activity (PASDAS ⩽ 3.2) and the absence of poor prognostic factors [C reactive protein (CRP) < 5 mg/dL, HAQ < 1, no radiographic erosions] were randomised open-label to either standard care with ‘step-up’ DMARD therapy or to symptomatic therapy with nonsteroidal anti-inflammatory drugs (NSAIDs) and local corticosteroid injections to inflamed joints. Key outcomes were the proportion of eligible cohort patients, consent and study completion rate.

**Results:**

Over the 15-month study period, only one eligible patient was randomised. Although oligoarthritis patients represented 45% of patients in this early PsA cohort, the majority did not have mild disease (24% raised CRP, 51% moderate disease activity, 13% radiographic damage and/or poor function). Of those meeting trial inclusion criteria, many patients refused treatment in the observational cohort prior to an invitation into the trial as they did not wish to be treated with DMARDs.

**Conclusion:**

The study was not feasible as designed. Oligoarthritis represents around half of initial PsA presentations, but the majority starting therapy have high-impact disease. A small proportion have mild oligoarticular disease but many are not keen on treatment with DMARDs, given the potential side effects of these medications. Further research is needed to support evidence-based treatment in this subgroup.

**Trial registration number:**

– ClinicalTrials.gov (NCT03797872) and EudraCT (2018-001085-42).

## Background

Psoriatic arthritis (PsA) is a highly heterogeneous form of inflammatory arthritis^1^ with a proportion of patients having mild nonprogressive disease.^2^ Well-validated prognostic factors in PsA can identify these patients including the number of active joints, systemic inflammation levels, radiographic damage and functional ability at presentation.^3–5^ However, there is little research addressing outcomes and treatment options for mild disease.^6–8^ The majority of phase 2/3 drug trials have required ⩾3 tender/swollen joints at baseline, but the mean joint counts in these studies are typically much higher.

Most physicians apply the same ‘step-up’ therapy to all patients supported by the recent European League Against Rheumatism (EULAR) treatment recommendations. These recommendations differentiate between polyarticular disease (where treatment is strongly recommended) and oligoarthritis (where treatment should be considered) but the general treatment approach is similar with conventional systemic disease-modifying antirheumatic drugs (csDMARDs) used as first line.^9^

It is likely that some patients are over treated with csDMARDs leading to unnecessary side effects for the patient and costs to the healthcare system. A previous study in undifferentiated peripheral spondyloarthritis (pSpA) found that 55% of patients did not require csDMARDs and could be managed with only intra-articular steroid injections and analgesia. However, only 4 of 59 patients with pSpA had a diagnosis of PsA.^6^

The aim of this study was to investigate the feasibility and acceptability of a study design to manage patients with mild PsA without using csDMARDs. This study was designed to enable the future design and power calculations for a definitive trial of delayed csDMARD treatment for mild PsA.

## Methods

### Trial design

**Figure 1.**
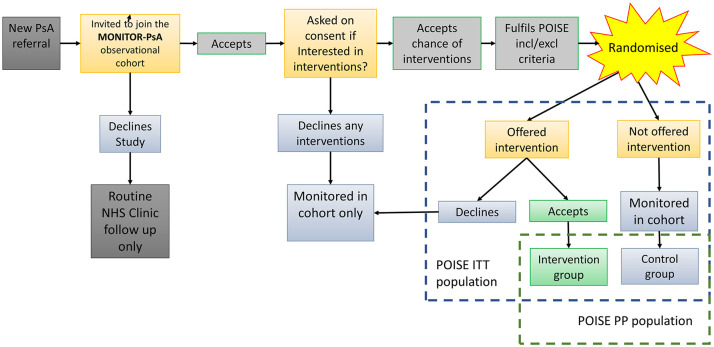
MONITOR-PsA and POISE randomisation and analysis schema.

The Psoriatic Oligoarthritis Intervention with Symptomatic thErapy (POISE) trial was a randomised open-label parallel group feasibility trial assessing the acceptability of conservative management in mild PsA and the feasibility of a future definitive trial. This feasibility study was established within the Multicentre ObservatioNal Initiative in Treat-to-target Outcomes in PsA (MONITOR-PsA) cohort, an inception PsA cohort recruiting at three centres in the United Kingdom (Oxford, Bath, Cambridge) at that time (NCT 03531073).^10^ This cohort recruits any patient seen in rheumatology departments with newly diagnosed PsA who has not yet had any disease-modifying treatment for this condition. It is a primarily observational study monitoring patients undergoing standard treatments for PsA. The study uses a Trials Within Cohorts (TWiCs) design.^11^ The TWiCs design embeds trials offering alternative treatment options within a cohort of patients having ‘treatment as usual’. All eligible patients recruited into the MONITOR-PsA study, who have consented to being approached about further research, are randomised either to remain in the cohort receiving treatment as usual or to the offer of an alternative treatment. They then choose whether to consent to this alternative therapeutic option (Figure 1). This feasibility study planned to recruit patients with oligoarthritis (⩽4 active joints) and the absence of poor prognostic factors and offer a delayed csDMARD treatment.

The principle objectives were to establish the proportion of patients within the MONITOR-PsA cohort eligible for and consenting to take part in the study; and to investigate what proportion of patients were not offered csDMARD therapy in the 48-week study period. Given the limited knowledge of the natural history of those with oligoarthritis, outcomes for both the standard care and intervention arm were required to inform the design of a future study. A maximum of 60 eligible participants from the MONITOR-PsA cohort were planned to be randomised 1:1 to receiving standard step-up DMARD therapy or symptomatic treatment. CONSORT guidelines were followed for reporting of this randomised feasibility trial.

## Participants

### MONITOR-PsA cohort eligibility

Eligible participants were adults (⩾18 years old) with a recent diagnosis of PsA (without restrictions on the duration of symptoms prior to diagnosis), recruited from the MONITOR-PsA observational cohort. Inclusion criteria for the MONITOR-PsA cohort are previously published^10^ and require a diagnosis of PsA confirmed by the ClasSification of Psoratic ARthritis (CASPAR) criteria,^12^ active PsA defined by ⩾ 1 tender or ⩾ 1 swollen joint or ⩾ 1 enthesis and no previous treatment with DMARDs for articular disease.

### Randomisation

As part of the consent process for the MONITOR-PsA Cohort, participants are asked to provide written consent to the following items as part of the Trials Within Cohorts design:

1. To be contacted by the research team about future interventional studies,
2. To be randomised by the research team for an invitation to participate in these future interventional studies, and
3. For anonymized data to be used as comparison as a control group for these future interventional studies.

If participants consented to the MONITOR-PsA cohort and to be contacted about future interventional studies and randomisation into these studies, then their baseline cohort data were reviewed to determine eligibility for the POISE interventional study. If eligible, participants underwent a first-stage randomisation either to remain in the MONITOR-PsA cohort as a control subject or to be offered the intervention arm. Participants randomised to standard care were treated within the cohort without any further information regarding this interventional study. If participants were randomised to be offered the intervention, a patient information leaflet on the POISE study was provided. Randomisation was confirmed in a second-stage process if participants provided written consent to the POISE intervention and all baseline investigations confirmed their full eligibility.

### POISE trial eligibility

The POISE study was approved by the South Central Research Ethics Committee Ref 18/SC/0261. The key inclusion criteria for the POISE study were the presence of oligoarthritis with ⩽4 tender/swollen peripheral joints and no poor prognostic factors. For safety reasons, as in standard care, baseline laboratory tests had to be within reasonable ranges to start csDMARD therapy (defined as haemoglobin count > 8.5 g/dL; white blood count (WBC) > 3.5 × 109/L; absolute neutrophil count (ANC) > 1.5 × 109/L; platelet count > 100 × 109/L; ALT and alkaline phosphatase levels < 3 × upper limit of normal) and participants receiving csDMARDs had to use adequate contraception.

Patients were ineligible for the POISE trial if they had

1. Any poor prognostic factors for PsA:
  - Raised C reactive protein (CRP, >4 g/dL)
  - Erosions on plain radiographs of the hands and feet
  - Health assessment questionnaire (HAQ) score > 1) or
2. Contraindications to nonsteroidal anti-inflammatory drugs.

When the study first opened, additional inclusion criteria were also in place, requiring patients to have low disease activity (PsA disease activity score or psoriatic arthritis disease activity score PASDAS ⩽ 3.2) and low impact of disease (PsA impact of disease or psoriatic arthritis impact of disease PsAID ⩽ 4). This was to minimise the potential for patients to be disadvantaged by delaying their treatment. However, these inclusion criteria were dropped part way through the study, with agreement with the trial steering committee, to improve recruitment (see results).

### Randomisation

Randomisation was undertaken via a centralised randomisation service and participants were randomised 1:1 to either continue in the cohort as part of the control group or to be offered symptomatic therapy in the intervention arm. The initial six participants (10% of the planned maximum number of randomisations) were to be allocated using a simple random list to seed the minimisation algorithm. Subsequent participants were to be allocated using a computer-generated randomisation algorithm using a minimisation approach (stratification factors: trial site, duration of disease prior to diagnosis) including a random element to ensure balanced allocations across the treatment groups. The random element was used to ensure that 80% of participants were allocated to the group that would maximise balance of stratification factors across the treatment arms, and 20% of participants were allocated to the other intervention to maintain unpredictability of the randomisation system.

The treatment received was open-label but clinical outcome assessments were performed by research staff blinded to treatment allocation within the MONITOR-PsA cohort for all participants.

### Interventions

Treatment in the control group was standard ‘step-up’ therapy (MONITOR-PsA Cohort study NCT03531073), which is defined by standard National Health Service (NHS) practice in these PsA clinics following current international recommendations^9^ and national requirements for the prescription of biologic therapy.^13–16^ While physician discretion is used, the most common initial therapy is methotrexate monotherapy, switching to alternative csDMARDs either alone or in combination and then biologic disease-modifying anti-rheumatic drugs (bDMARD) therapy in cases of nonresponse.

For patients randomised to symptomatic therapy, treatment with standard care csDMARDs was not commenced and instead local administration of glucocorticoid injections (methylprednisolone or traimcinalone) to affected joints with concomitant oral nonsteroidal anti-inflammatory drugs (NSAIDs) were offered as indicated to manage symptoms. All active joints were injected or glucocorticoids could be given by IM injection if multiple joints were involved and the patient declined multiple injections.

All participants in both groups were to be reviewed every 12 weeks and were instructed to contact the research team if their disease flared between these visits, in order to facilitate an interim review. If any joint required more than two local injections of glucocorticoid within a 6-month period, the patient was deemed to have failed symptomatic therapy and was to be withdrawn from symptomatic therapy and be treated as per usual care (in most cases with csDMARD therapy). If participants required csDMARD therapy, they were to be offered rescue therapy as per usual clinical care but were to be asked to continue with data collection for the trial. This ensured that sufficient data could be collected for the trial while risks in delaying treatment to the individual were mitigated.

### Data collection and outcomes

Baseline assessments were performed within the MONITOR-PsA cohort prior to randomisation. Clinical assessment of disease activity was performed by the research team including 68/66 tender/swollen joint counts, enthesitis and dactylitis counts, psoriasis area and severity index (PASI) and body surface area (BSA) of psoriasis. Patient-reported outcomes included assessments of global disease activity, pain, health assessment questionnaire (HAQ), PsAID, short form (SF-36), EQ5D-5 L, work productivity and activity impairment (WPAI), Bath ankylosing spondylitis disease activity index (BASDAI), Bath ankylosing spondylitis functional index (BASFI). In line with the cohort, trial participants were to undergo the same assessments again at 12, 24, 36 and 48 weeks.

In addition, participants randomised to the POISE intervention arm were also to be asked to have a baseline ultrasound (US) scan of key joints and entheses to establish subclinical inflammation and see if it may identify a subgroup of patients for whom conservative treatment is most beneficial. A baseline ultrasound of 44 joints and 10 entheses was to be performed as the optimal sites for US in PsA were not yet established.

### Sample size

As a feasibility study, one of our key outcomes was the recruitment rate itself. Publications suggest a sample size of 12–30 patients per arm for a feasibility study^17,18^ and therefore a maximum of 60 participants were to be randomised in this study in a 1:1 ratio.

### Statistical methods

The primary outcome of the study was to assess feasibility by assessing the proportions of

- eligible participants in the cohort over the recruitment period for POISE,
- eligible participants consenting to participate in the POISE trial, and
- participants requiring escalation to DMARD therapy within the first 48 weeks.

The first two objectives were planned to be addressed by examining eligible participants over the entire study period and eligible participants/recruitment per month. The third objective was planned to be examined by calculating the proportion of participants randomised to the symptomatic therapy intervention arm, who had met the criteria for escape to DMARD therapy during the 48-week study period.

A future, definitive trial would be an independent study that would not reuse data from these participants. However, the data from this feasibility study would allow us to examine the descriptive statistics around the different outcome measures, establish sample size and to plan which outcomes to include as primary and secondary key outcomes.

The primary outcome of a future proposed trial was chosen as the proportion of participants maintaining low disease activity as measured by the PASDAS (⩽3.2) at week 48. The PASDAS is a composite score including both clinical assessment and patient-reported outcomes and has been validated in oligoarticular disease. It is calculated as (((0.18√physician global visual analogue scale (VAS)) + (0.159√patient global VAS) – (0.253 × √short form 36 physical component score (SF36-PCS)) + (0.101 × log (natural log) (LN (swollen joint count (SJC + 1)) + (0.048 × LN (tender joint count (TJC) + 1)) + (0.23 × LN (Leeds enthesitis index + 1)) + (0.37 LN (tender dactylitis count + 1)) + (0.102 × LN (C-reactive protein (CRP )+ 1)) + 2) × 1.5.^19^ Descriptive data on the PASDAS were planned to be collected in this feasibility study to allow for estimation of appropriate sample size for any future definitive trial.

Reported tolerance, particularly those side effects related to treatments would be presented for both groups. In addition, reasons for nonconsent and any suggestions for improving the study design as well as the feasibility of outcomes were to be considered when designing any future definitive trial.

## Results

The MONITOR-PsA cohort opened to recruitment in Oxford on 12 April 2018 and in Bath on 22 October 2018. The POISE trial opened on 17 April 2019 in Oxford and 19 September 2019 in Bath. During this setup phase, a review of potential patients that were eligible in the MONITOR-PsA cohort was performed in January 2019. This review showed that 13 of 37 (35%) patients recruited to that date had presented with oligoarthritis but only 5 had no poor prognostic markers. While three of these met the PsAID criteria, none of them had a PASDAS score ⩽ 3.2.

At a trial management meeting, clinicians and patient partners offered their insight and suggested the removal of the PASDAS and PsAID inclusion criteria to improve recruitment. The patients felt that this was acceptable as more patients would be offered the trial intervention, but would be able to decline this if they felt that their disease burden was too high. This suggestion was reviewed by the trial steering committee who suggested a 3-month trial period when opening the study but with a plan to alter the inclusion criteria after that period if required. This revised protocol 6.0 was approved and implemented on 16 October 2019.

The study remained open until 16 July 2020 as planned. During this period, only one patient was recruited and that patient was randomised to the standard care arm. They started on csDMARD therapy but were lost to follow-up shortly after and upon review of their clinical notes, appear to have stopped treatment with csDMARDs of their own accord. No serious adverse events were reported for this participant before their withdrawal.

The first aim of the POISE study was to establish how many eligible participants were identified in the cohort over the recruitment period for POISE. Given the failure to recruit, we have investigated the potential eligible participants in the entire MONITOR-PsA cohort. This is shown in Table 1, which highlights that while oligoarthritis is relatively common, identifying patients with no poor prognostic factors and low impact of disease is uncommon. Only 4% of the MONITOR-PsA patient population were deemed eligible. More patients would have been eligible with the change in protocol inclusion criteria in October 2019, but the numbers were still low (Table 2, 9% of the MONITOR-PsA patient population).

**Table 1.** Participants meeting protocol 1.0 inclusion criteria (oligoarthritis, no poor prognostic factors, PASDAS ⩽ 3.2, PsAID < 4) for the trial.

	Bath	Cambridge	Oxford	Total
Patient screened (i.e. entered into MONITOR-PsA even before POISE trial was opened) (n)	17	36	68	121
Exclusion criteria^a^				
5 or more active joints (n)	4	22	40	66
CRP > 4 (n)	4	7	2	13
PASDAS ⩽ 3.2 (n)	5	0	23	28
Poor prognostic marker^b^ (n)	2	5	0	7
Not consented to additional studies (n)	1	0	0	1
Total ineligible for version 1.0 (n)	16	34	65	115
Proportion ineligible (%)	94	94	96	95
(Potentially) eligible (n)	1	1	2	4
Included in POISE (n)	0	0	1	1
Proportion potentially eligible (including participant in POISE) (%)	6	3	4	4
Missing data (n)		1		1

CRP, C reactive protein; MONITOR-PsA, Multicentre ObservatioNal Initiative in Treat-to-target Outcomes in psoriatic arthritis; PASDAS, psoriatic arthritis disease activity score; POISE, Psoriatic Oligoarthritis Intervention with Symptomatic thErapy; PsAID, psoriatic arthritis impact of disease.

a Exclusion criteria mutually exclusive; primary identified reason listed for each participant.

b Includes radiographic damage and/or HAQ > 1.

**Table 2.** Participants meeting protocol 6.0 inclusion criteria (oligoarthritis, no poor prognostic factors, PASDAS and PsAID removed as inclusion criteria) for the trial.

	Bath	Cambridge	Oxford	Total
patient screened (i.e. entered into MONITOR-PsA even before POISE trial was opened) (n)	17	36	68	121
Exclusion criteria^a^				
5 or more active joints, n	4	22	40	66
CRP > 4, n	4	7	15	26
Poor prognostic marker^b^, n	4	5	3	12
Pregnant, n	1			1
White blood count (WBC) > 3.5 × 109/L, n	1			1
Not consented to additional studies, n	2			2
Total ineligible for version 6.0, n	16	34	58	108
Proportion ineligible, %	94	94	85	89
(Potentially) eligible, n	1	1	9	11
In POISE, n	0	0	1	1
Proportion potentially eligible (including participant in POISE) (%)	6	3	13	9
No info		1		1

CRP, C reactive protein; MONITOR-PsA, Multicentre ObservatioNal Initiative in Treat-to-target Outcomes in psoriatic arthritis; POISE, Psoriatic Oligoarthritis Intervention with Symptomatic thErapy.

a Exclusion criteria mutually exclusive; primary identified reason listed for each participant.

b Includes radiographic damage and/or HAQ > 1.

Anecdotally, investigators involved in the MONITOR-PsA cohort reported that a number of patients with mild PsA, often oligoarthritis, were not happy to accept treatment with csDMARDs, and therefore unable to join the MONITOR-PsA cohort. This reluctance to consider csDMARDs became more marked during the 2020 COVID-19 pandemic; however, was an issue prior to this. Patients felt that their disease was mild and did not warrant the use of regular medication. In these situations, patients were routinely discharged back to primary care and therefore not included in the MONITOR-PsA cohort. Despite considering all the patients potentially eligible within the MONITOR-PsA cohort, there was a selection bias in recruitment for those willing to consider systemic therapy.

During the study, only one eligible participant was identified to be included in the POISE trial but as they were randomised to the standard care arm, they did not undergo the consent process for the POISE trial. We are therefore unable to conclude how many patients would have consented when offered the symptomatic therapy intervention. Given the anecdotal feedback above, it seems that this intervention would be accepted by participants; however, they may not have felt equipoise was present when considering the two treatment arms. Most participants had strong views either for or against csDMARD therapy at the time of diagnosis. As no patients were recruited to the intervention arm, we are unable to calculate the proportion of participants requiring escalation to DMARD therapy within the first 48 weeks.

## Discussion

The aim of this study was to establish the feasibility of delaying csDMARD use in mild PsA. Previous research has suggested that patients with oligoarticular peripheral SpA may not require DMARD therapy and therefore there is potential to manage mild PsA without DMARDs, thus minimising potential side effects for individuals and incurring cost savings for the NHS. However, this feasibility study only recruited one participant during the planned recruitment period, confirming that this current design is not feasible. The study had significant input from patient research partners with PsA to try to ensure that the intervention was appropriate and acceptable to patients, but despite this, failed to recruit.

Due to the TWiCs study design, it was planned that participants would first be recruited to the MONITOR-PsA cohort and be routinely treated with csDMARD therapy and then eligible participants would be randomised to the offer of the POISE intervention. With support from our patient research partners, we believed that the POISE intervention would have been acceptable to eligible patients; however, we did not foresee that many potential participants would decline DMARD therapy at the time of diagnosis and therefore would never be included in the MONITOR-PsA study.

MONITOR-PsA site investigators have reported that these potential participants, patients with mild PsA characterised by low joint counts and disease impact, often had strong views on avoiding DMARD therapy at diagnosis and therefore were not eligible for inclusion. Even if this had been a traditional randomised control trial (RCT) design where the study was offered to patients without prior enrolment in the cohort, investigators felt that many of these patients would have declined. In this case, it was not the POISE intervention that they found unacceptable, but the ‘standard’ treatment with csDMARDs. This concern around medications has been identified in previous qualitative work in PsA^20^ and within the ongoing James Lind Alliance priority setting partnership in PsA (L Coates, data on file).

This study highlights an ongoing unmet need in mild PsA that current research studies have not addressed appropriately for patients. Future research addressing mild PsA needs to include strong patient representation specific to this patient group, as most patient representatives in local, national and international research committees have taken DMARD therapy and may hold different views to those with mild disease. Observational data on patients managed without csDMARD therapy may be useful to establish prognostic markers and aid identification of those who require DMARDs and guide treatment strategy. Future studies addressing mild disease may need to consider that these patients are often not willing to take csDMARDs and may not remain under routine rheumatology follow-up. Alternative study strategies like remote follow-up may be helpful in this group. Extending recruitment to clinics outside rheumatology may be beneficial, for example, screening for arthritis within dermatology or primary care clinics. The data reported here on the MONITOR cohort also highlights that although oligoarthritis is common at presentation, ‘mild’ disease in ongoing cohorts may be much less common when full examination and assessment of prognostic markers is made. Patients with more severe or impactful oligoarthritis also represent an underresearched subgroup of PsA.

Psoriatic arthritis is well recognised as a heterogeneous condition and modern PsA patient cohorts highlight that over half of patients initially present with oligoarthritis (<4 joints involved).^21^ Within oligoarticular PsA, there is a wide variety of disease severity but all of these patients represent an unmet need in terms of optimal treatment strategy.^22,23^ Treatment choice for those with oligoarthritis cannot be evidence based as nearly all large drug trials in PsA require a minimum of three tender and swollen joints, and routinely recruit a high proportion of polyarticular patients with average joint counts over ten. There has been very little research into those with mild oligoarthritis, as these patients have not been perceived to be in urgent need and may not be routinely monitored in rheumatology clinics. Future research is required to support clinicians and patients in establishing their individual prognosis and in enabling personalised treatment strategies in this common subtype of PsA.

Trial protocol available from the study team on request.
