# Erratum to “Clinical effectiveness of symptomatic therapy compared with standard step-up care for the treatment of low-impact psoriatic oligoarthritis: the two-arm parallel group randomised POISE feasibility study”

**DOI:** 10.1177/1759720X221077827

**Published:** 2022-02-09

Rombach, I., Tucker, L., Tillett, W., et al. Clinical effectiveness of symptomatic therapy compared with standard step-up care for the treatment of low-impact psoriatic oligoarthritis: the two-arm parallel group randomised POISE feasibility study. Ther Adv Musculoskelet Dis 2021; 13: 1-10. https://doi.org/10.1177/1759720X211057668

In the initial online version of the article, copyright year and volume number were incorrect. The correct copyright year is 2022 and Volume 14. The online version of the article has been corrected.

